# The need to modify physical activity messages to better speak to older African American women: a pilot study

**DOI:** 10.1186/s12889-015-2317-x

**Published:** 2015-09-25

**Authors:** Emerson Sebastião, Wojtek Chodzko-Zajko, Andiara Schwingel

**Affiliations:** Aging and Diversity Lab, Department of Kinesiology and Community Health, University of Illinois at Urbana-Champaign, Huff Hall, 1206S. Fourth St, Champaign, IL 61820 USA

**Keywords:** Physical activity, African American, Older adults, Health promotion, Health communication, Focus groups

## Abstract

**Background:**

Combating the physical inactivity crisis and improving health and quality of life is a challenge and a public health priority, especially in underserved populations. A key role of public health consists of informing, educating, and empowering individuals and communities about health issues. Researchers have found that mass communication messages often have limited effectiveness in reaching and impacting the health of underserved populations. The present pilot study was designed to explore perceptions of older African American women (AAW) in response to widely disseminated public information pertaining to physical activity (PA) and aging.

**Methods:**

A total of 10 older AAW aged 60 years and over participated in this study. Participants were evenly assigned in one of the 2 focus groups (i.e. active, *n* = 5; and inactive, *n* = 5) based on their PA level. The focus group approach was employed to gather information about widely available public information materials related to PA that target the adult and older adult population. The three guides used were: (1) Exercise and Physical Activity: Your Everyday Guide; (2) The Physical Activity Guidelines for Older Adults; and (3) Be Active Your Way: A Guide for Adults. NVIVO 10 software was used to help in the qualitative data analysis. Descriptive thematic analysis was employed in identifying, analyzing and reporting patterns/themes within the data.

**Results:**

Older AAW in the present study identified some shortcomings in current public health materials. Participants from both focus groups raised concerns regarding language and the types of activities used as examples in the materials. After analysis, two themes emerged: “We may have trouble in reading it” and “It does not reflect us”. Participants’ evaluation was found to be similar between the active and inactive focus groups.

**Conclusions:**

Older AAW’s perceptions of the materials suggest that materials intended to educate and motivate the general public towards PA need to be modified to better speak to older African American women, especially to those who are sedentary and have difficulty in building PA into their daily lives.

## Background

Combating the physical inactivity crisis and improving health and quality of life is a challenge and a public health priority, especially in underserved populations. The rates of preventable chronic diseases are disproportionally high among African Americans and could be reduced through physical activity (PA) and other health behavior interventions. In the United States, older African American women (AAW; those aged 60 years and over) are particularly prone for physical inactivity [1, 2] and resultant chronic diseases [3–5]. Studies exploring barriers to and motivators for PA participation in African Americans have found a variety of personal, interpersonal, and environmental factors influence PA behavior in this population [6–9]. Factors negatively impacting PA include but it is not limited to lack of time, health concerns, knowledge, family responsibilities, and lack of social support [7, 9]. Factors positively impacting PA behavior include but it is not limited to health, availability of community resources, green areas nearby, and pursuit of better health [6–8]. Promoting PA among underserved groups requires a concerted effort to identify factors that are specific to a particular group in order to develop the most effective culturally relevant strategy to increase PA in this population and help mitigating ongoing PA-related disparities [10].

A key role of public health consists of informing, educating, and empowering individuals and communities about health issues [11]. Educating the general population about PA and other health behaviors necessitates the development and dissemination of a wide variety of educational materials [12, 13]; Educational materials regarding PA, for example, normally take the format of internet-based information (i.e. websites), brochures, pamphlets and booklets. These materials are developed not only to inform about the amount and intensity of PA necessary to achieve health benefits, but to provide information about the benefits of PA for the prevention, management, and treatment of certain conditions, as well as to provide examples of specific PA that might be favored by particular populations, etc. During the development process for the community health messages assessed in the present study, the Centers for Disease Control and Prevention adopted both subject matter experts and community members during the process of testing different messages [14]. However, the degree to which the public health messages/materials used to promote PA were tested on sufficiently diverse subsets of the population (i.e. minorities, women, and older adults) is unclear. Therefore, it is possible that well-intended PA messages may not be clearly interpreted by, or may not be reaching older AAW, a sub-group that is at a particularly high risk for inactivity and the related chronic disease and conditions. This concern is reinforced by previous research that has shown that older AAW are not aware of the amount of PA that is needed to maintain or improve health and well-being [6]. In addition, other researchers have found that mass health communication messages have been shown to have limited effectiveness in reaching and impacting the health of underserved populations [15].

Health communication messages play a critical role in public health and constitute an important means to promote healthy behaviors by educating and empowering people through increasing knowledge and understanding. In the past, public health messages have often been developed by health professionals with limited experience in mass communication [16, 17]. This has sometimes resulted in ineffective communication and a limited ability to raise public awareness of the importance and benefits of having a physically active lifestyle [18]. More recently, public health has begun focusing on developing materials explicitly designed to educate and assist lay audiences to pursue a healthier lifestyle [19, 20]. In the context of PA, leading public health agencies such the Centers for Disease Control and Prevention and the National Institute on Aging along with the Department of Health and Human Services have played an important role in the development and dissemination of public information materials. Examples of such materials include; Exercise and Physical Activity: Your Everyday Guide from the National Institute on Aging [19]; the Physical Activity Guidelines for Americans [21]; and Be Active Your Way: A Guide for Adults [22].

Research into health communication has noted the critical role played by culture and cultural values in effective health communication [23]. Effective communication is not restricted to the recognition of cultural values but belief systems, religion, life experiences, and group identity may also work as powerful filters through which information is received [23]. African Americans are a group that have singular identity, culture and beliefs [24] that distinguish them from other racial/ethnic groups in the United States, due in part to their history and legacy of adverse events in this country [25, 26]. Messages and materials developed to promote healthy lifestyles, such as PA, are designed to increase PA levels and ultimately to improve health and consequently quality of life. However, studies show that older AAW have one of the lowest rates of PA participation at recommended levels [1, 27]; and that mass health communication messages have shown to have limited effectiveness in reaching and impacting this population [15].

Although there is some evidence suggesting that tailored messages are likely to be viewed as more relevant than more generic communications on behavior change [28], the conclusions for that derived from narrative review methods. In the view of some researchers, narrative review methods have significant shortcomings [24]. Moreover, to the best of our knowledge, no study has attempted to explore how older AAW perceive government sponsored and developed materials used to promote PA for older adults. Public health has invested significant time and resources in creating and disseminating informative materials as a mean to promote PA at the population level. It is important that the impact of these materials be sufficient to warrant the investment. The present pilot study was designed to explore perceptions and reactions of older AAW to widely disseminated public information pertaining to PA promotion and aging. In doing so, this pilot study will add to the current literature by providing preliminary new insights on how PA-related informational materials are perceived by one of the least physically active population groups.

## Methods

The current pilot study incorporated focus group discussions with older AAW along with a simple quantitative analysis to gather information about widely available public information materials related to PA. We decided to use focus group because of their ability to provide an in-depth understanding of the issues to be addressed in this study. By asking groups of older adults with similar characteristics to discuss their perceptions and beliefs we were able to gain richer and more detailed information than could have been obtained through individual interviews alone [29]. Prior research [29, 30] has shown that valuable lessons can be learned by asking questions such as: What, why, do you think, etc. As a pilot study, our main interest was to let participants talk and discuss as much as possible (i.e. brain storming). In this sense, the guide questions used for this study were simple, however, at the same time, with the potential to collect important information to help us achieve the purpose of the study. Table 1 displays in detail the guiding questions used during both focus group discussions.

**Table 1:** Interview guide used during focus group discussion of materials used to promote physical activity among the adult and older adult population

Guide questions	Description
1	What are the brochures talking about?
2	How did you feel after read these messages?
3	Are the messages clear to you? Why or why not?
4	Did anyone feel the messages are somehow confusing? Difficult to understand? Why?
5	Which of those do you relate to more? Why?
6	Now think about older African American women in general. Would they be able to understand the message? Do you think your peers would have difficult in reading it/understanding it?
7	What are some of the things we need to change, if any, in order to make the message clear to the African American community?
8	What do you think about this document? Have you ever seen it before? Does the information included motivate you? Does it catch your attention? What makes you interested in reading it or not interested in reading it [discussion of one document at a time].

### Participants

Participants were recruited from a previous study that was conducted to examine PA levels in older AAW. A total of 20 older AAW participated in that study and had their PA level measured objectively through accelerometers (Actigraph GT3x plus). Habitual weekly PA was acquired after participants’ wore the accelerometers for 7 consecutive days and the data were analyzed using Actilife 6 software. The volume of moderate to vigorous physical activity (MVPA) was determined using Freedson and colleagues [31] cut-points (≥1952 counts per minute). In our previous study, 9 women were classified as physically active according to current aerobic public health recommendations (≥150 min of MVPA per week), and 11 were classified as inactive (<150 min of MVPA per week) [32]. Based on that sample and its distribution of active and inactive women, we drew our sample for the current pilot study. Due to the pilot nature of the study, our concern was primarily the identification of general perceptions and no attempt was made to assess the degree of saturation achieved.

A total of 10 older AAW (active *n* = 5; and inactive *n* = 5) aged 60–80 years living in a central Illinois community were selected from the database of the mentioned study. PA status was used a criterion for focus groups assignment. Thus, active women were assigned into the active focus group and inactive women assigned into the inactive focus group. It was important to include both active and inactive focus groups because perceptions about the materials could vary between those women who meet the current guidelines for PA and those who do not. Active women may have had prior access to the materials. Therefore, using two groups, would allow us to identify other variables such as whether and how they had prior access to the materials.

### Data collection and analysis

Data were collected during March and April of 2014. Demographic, socioeconomic and health information were collected using a questionnaire. Information on age, marital status, income, weight, height, Body Mass Index (BMI), history of chronic diseases, and medications were collected.

Focus groups were conducted in 2014 by an African-descendent trained facilitator with experience in conducting qualitative studies (individual interviews and focus group) and who has more than 8 years of experience in the study of PA, health and aging. The focus groups took place at the University of Illinois at Urbana-Champaign where this study was based, and each lasted approximately 60–75 min (i.e. recorded interview portion). Only participants and the facilitator were in the room during the focus groups. Materials (brochures) used by leading public health agencies to promote PA and exercise were presented to each group and participants were asked to comment on each of them. The materials presented during the focus groups were: (1) Exercise and Physical Activity: Your Everyday Guide from the National Institute on Aging [18]; (2) The Physical Activity Guidelines for Older Adults retrieved from the Centers for Disease Control and Prevention (CDC) website [20]; (3) Be Active Your Way: A Guide for Adults from the Department of Health and Human Services [21]. All materials were presented in the format of a brochure. Each participant received an envelope containing a hard copy of the 3 brochures. Although a variety of materials related to PA exist, the reasons for choosing this set of materials lie in the fact that they were developed by leading public health agencies in the United States (i.e. Centers for Disease Control and Prevention, National Institute on Aging, Department of Health and Human Services). Moreover, they are the newest materials available designed for educating the lay population of adults and older adults. Before engaging in the discussion of the materials, participants were given approximately 30 min to browse into the brochures provided. After the 30 min, each participant was asked to subjectively rate individually each of the 3 brochures on a scale from 1 to 5 in terms of clarity and content, 1 being poor and 5 being excellent. Following the brochures’ evaluation, the focus group discussion commenced and was audio-recorded and later transcribed verbatim by a native English speaker for analysis. Before analysis, the research team validated the transcribed documents by checking it against the audio-record file.

Sociodemographic, health, physical activity data, and the evaluation scores reported for the brochures’ were analyzed using mean and standard deviation. Additionally, *t* tests were used to compare PA level (i.e. levels of MVPA) between active and inactive groups. The Mann Whitney *U*-test was used to compare the mean score of the evaluation for each brochure between groups. Statistical significance was set at *p* <0.05.

Data from the focus group discussions were analyzed using a descriptive thematic analysis approach based on realist and semantic methods, adopting the six phases described by Braun and Clarke [33]. Two native English speakers with experience in transcribing interviews were hired to transcribe all interviews. Transcriptions were then checked for accuracy by the investigators, and analyzed using thematic analysis. The thematic analysis involved the identification of themes that emerged from the data as being important to the description of the phenomenon. It involved the process of identifying, analyzing, interpreting, and reporting the themes. Initially, each of the three researchers involved in the study separately coded and analyzed the data using NVIVO 10 software [34] to look for themes. Subsequently, the researchers compared codes and themes for agreement, retaining only the themes that reached an agreement. When negative cases arose, the researchers discussed each case, using them as an opportunity to further refine each theme.

Credibility and reliability of the data were examined employing prolonged engagement and triangulation between three researchers. Prolonged engagement was achieved by spending adequate time with participants during the interview process, exploring participants’ perceptions about the subject matter of the study. Moreover, the facilitator is engaged with members of the African American community (both young and older adults) of Urbana-Champaign area, which helped to build trust. Such procedure helped in detecting and accounting for distortions that might contaminate the data. Triangulation was also used to ensure data reliability and credibility. In this part of data analysis, the three investigators reviewed the data and produce codes and themes. After this stage, investigators met to check and discuss similarities and differences in codes and themes produced before producing the final report.

### Ethics

The Institutional Review Board of the University of Illinois approved the research protocol of the present study (IRB# 13309), and a signed informed consent was obtained from all participants before data collection.

## Results

Detailed characteristics of the sample are given in Table 2. Participants from both groups present similar demographic and health characteristics. In terms of PA level, the average minutes per week of MVPA of the active group was significantly higher compared to the inactive group (288.9 ± 82.7 vs 127.6 ± 19.2; t(8) = −4.249; *p* = 0.003).

**Table 2:** Sociodemographic and health characteristics of the participants

	Total	Active focus group	Inactive focus group
	(*n* = 10)	(*n* = 5)	(*n* = 5)
Age (mean, SD)	66.7 (5.98)	64 (4.85)	69.4 (6.23)
Education (n)			
High School	2	–	2
Some College	3	3	–
College or more	5	2	3
Marital status (n)			
Married	5	3	2
Divorced/Separated or widowed	5	2	3
Engaged in any religion (n)			
Yes	10	5	5
Children (n)			
Yes	9	5	4
Living status (n)			
Alone	3	1	2
Someone live with me	7	4	3
Income (n)			
$ 20,000 – $ 29,999	1	1	–
$ 30,000 – $ 39,999	4	–	4
$ 40,000 – $ 49,999	1	1	–
$ 50,000 and over	4	3	1
Self-reported health (n)			
Good	6	3	3
Very good	4	2	2
Do you believe that one can improve health though exercise, balance diet, and stop smoking? (n)			
Yes	10	5	5
Chronic disease (n)			
0	3	2	1
1 – 2	4	3	1
≥3	3	–	3
Medication (n)			
0	4	3	1
1 – 2	4	2	2
≥3	2	–	2
Health Insurance (n)			
Yes	9	4	5
BMI (Kg/m^2^)	29.48 (3.84)	28.48 (4.07)	30.48 (3.76)
Difficult in ADL or IADL (n)			
Yes^a^	1	–	1
No	9	5	4

*BMI* Body mass index

^a^Participant reported some difficult in walking

The quantitative procedure adopted to score the materials along with the qualitative data gathered about the materials revealed that participants’ evaluation on the PA and exercise materials was found to be similar between the active and inactive focus group. The following table summarizes the evaluation made by the participants in each group for each of the three sets of materials.

It was observed that the brochure “Exercise and Physical Activity” brochure was perceived to be the best in terms of clarity and content followed by the “Be Active Your Way” brochure. Participant scores for the two mentioned brochures may reflect their perception of the simple language and abundant illustrations and the fact that the information is presented concisely and in a step-by-step manner. A quote from a participant in the sedentary group best describes the women’s preference for the mentioned materials:

- *“… they are easy to read, they are easy to comprehend and read. The one NIH book it’s more visible and it is more compact and it gives you all the instructions that you need… The be active sheet, it’s compact and it doesn’t give you a lot but it gives you enough to get started… they even tell you how to make it safe, reduce risks, injuries… I think every African American home should have one of these.” (66 years - Inactive)*

### Thematic analysis

Despite expressing a positive impression regarding two of the three materials presented, some issues regarding language and types of activities used as examples in the materials emerged during both focus group discussions. After analysis, two themes emerged: “We may have trouble in reading it” and “It does not reflect us.”

#### We may have trouble in reading it!

Both language and visual imagery emerged as important in both groups. Participants talked about how the information retrieved from the Center for Disease Control and Prevention (CDC; Physical Activity Guidelines for older adults) are confusing and challenging because of the difficult language, technical terminology, and information overload. The following is an example;

- *“… Not thought-out, but some people might not know what moderate is, you know, to intensity… because you do have a lot of what you call the average Joe, they’re not middle class, they’re not gone to college, half of them really basically have not even finished high school… people like that, which is call the average Joe, you are going to have to do – break it down in just a little bit more, what they said, layman’s terms…” (61 years - Active)*
- *“… the words, need to be brought down to another level, I think that some of it could be a little elevated for some people… if the seniors, they may be having problems with the reading… so they can’t relate or read all those words…some African American descendent would have trouble with this… it’s way too much information, it’s information overloaded…” (72 years - Inactive)*

The concern pointed out by the groups in the CDC message appeared to be less evident in the “Be Active Your Way” brochure. Participants from both groups stated that the document was enhanced by making the message more compact, more visual and by providing some examples that may help them to better understand some of the technical terms used. The following is an example;

- *“… if you’re interested in the questions you know I can just read this little snippet here, I don’t have to read the whole document… this sheet is more compact and it doesn’t have as much information… it gives you a few brief things… it has the vigorous activities and the moderate activities which you can identify with…” (73 years - Inactive)*
- *“… It’s a little bit more condensed than the CDC paper and it doesn’t give you what the, all the wordings that the CDC paper does… it’s a little bit easier to read…” (66 years - Inactive)*
- *“… It’s more colorful, it has perked my interest. I’m a visual learner… I like just the bar on the bottom that says, “Be active, healthy, and happy”. I think those are active words… I want to be active, healthy, and happy… (66 years - active)*

However, one participant in the sedentary group still expressed some concerns about the use of technical terms in the document, for example stating:

- *“… you have to be knowing what some of these words means… muscle strengthening… aerobic…, cause everybody don’t know what aerobics is… a lot of people take a different connotation than what it is.” (67 years - Inactive)*

In contrast, language was mentioned only in a positive context by the participants of both groups with regard to the “Exercise and Physical Activity” brochure. Women commented on how accessible the message is by talking about the easy-to-read format that appeared, the balance between visual illustrations and instructions and detailed explanations. The following is an example;

- *“… I think that the instructions for the exercises are easy to read… they basically word it easy… and them of course having the illustration, kind of step by step, helps because you can actually see a picture and associated with the instructions you have… I like it because they have actually broken it down… they go in-depth, thinking about beyond a certain class of people… and make them want to read…” (65 years - Active)*
- *“… So they packaged it well, and as you begin to read, the language, not only the pictures, gets you a little bit interested in it… In addition I noticed the charts, the logs, the records… and I liked the demonstrations on how to do it… it breaks it all down for you… it explains to you why you’re doing these exercises…” (78 years - Inactive)*

#### It does not reflect us!

Despite acknowledging the fact that examples of activities helped them better understand some technical terms, the women in both groups were concerned about the examples provided in the materials. The women mentioned that many of the examples described in the materials do not reflect the older African American population. The following are examples;

- *“…for black women… there were restrictions on the physical activity for black Americans… You couldn’t even go swimming in the pool. You were restricted. You certainly couldn’t hang out at the track in the high school and walk around the track at a high school, and be suspect. You couldn’t go to a public beach and swim…” (65 years - Active)*
- *“… it has a lot to do with economics… looking at these lists* [examples of activities provided in the materials for moderate and vigorous intensity] *to me I still keep going back to the black versus white, whereas these are not the things we have thought of all of our entire lives.” (66 years - Inactive)*

A summary of the themes that emerged from the focus groups discussion is given in Table 4.

## Discussion

This pilot study was designed to explore perceptions and reactions of older AAW to widely disseminated public information pertaining to PA and aging. Regular PA is one of the most affordable means to achieve health and to prevent chronic disease [35, 36]. Improving the health outcomes of African Americans is a public health priority and a step towards mitigating health disparities [37, 38]. Although the body of evidence about factors influencing PA in African Americans women (adult and older adult) has grown [9, 39–41], very little research has been done to explore the effectiveness of public health information pertaining to PA and aging in reaching older AAW and motivating them to be more physically active. The findings of this study suggest that public health professionals should expand their use of health communication strategies when designing materials that target under-represented groups [15]. However, to design effective interventions it is important to work collaboratively with communities experiencing problems to overcome a historical context of distrust and to create meaningful, effective health communication strategies.

Quantitatively, no significant difference was observed between groups when comparing the average score of the evaluation for each of the materials taking into account clarity and content (refer to Table 3). Both groups appeared to agree that the PA guidelines brochure downloaded from the CDC website in its current form is not appealing to the African American community, especially older AAW. On the other end, they agreed that the Exercise and Physical Activity: Your Everyday Guide may motivate them to be more active (refer to Table 4 – theme 1). This demonstrates that regardless PA status; both active and inactive women in this study perceive PA-promoting materials in similar way. Although active older AAW may have found other ways to motivate themselves to be more active, better designed materials are important to help inactive women to build PA into their daily life. The present study identifies some shortcomings in public health materials aimed to promote PA in the adult and older adult population. In terms of comprehension, the findings show that 2 out of the 3 materials (i.e. Exercise and Physical Activity: Your Everyday Guide and Be Active Your Way: A Guide for Adults) used in the focus group discussions are considered to be fairly easy to understand in the perception of the participants (refer to Table 4 – theme 1). In addition, imagery seems to be a concern in the materials reviewed. Our findings suggest that an inability to visualize themselves carrying out the activities described may be barriers faced by older AAW (refer to Table 4 – theme 2). Together, these factors may be working as a barrier that detracts from an individual’s understanding and appreciation of the content. Since these informational materials are used to inform the general public about PA and exercise and to empower them through knowledge, the purpose may not be being achieved.

**Table 3:** Participants’ evaluations on three different materials used to promote physical activity among adults and older adults in terms of clarity and content. Information presented as mean and standard deviation

Material	Active focus group	Sedentary focus group	p-value*
	(*n* = 5)	(*n* = 5)	
Physical activity guidelines	2.1 (.74)	2 (0.70)	.841
Be active your way	3.4 (.42)	3.8 (.83)	.548
Exercise and physical activity	4.7 (.27)	5 (0)	.151

Scale: (1 = poor and 5 = excellent); *p-value for Mann Whitney *U*-test

**Table 4:** Summary of the themes that emerged during the focus groups discussion on materials pertaining to physical activity promotion and aging

Theme	Brief theme description	Sample quotes
1. We may have trouble in reading it	This theme represents the perceptions of older African American women towards the PA materials presented to them during the focus group discussion. Perceptions varied among the three materials.	**Physical Activity Guidelines for Older Adults:** *“… the words, need to be brought down to another level, I think that some of it could be a little elevated for some people… if the seniors, they may be having problems with the reading… so they can’t relate or read all those words…some African American descendent would have trouble with this… it’s way too much information, it’s information overloaded…” (72 years - Inactive)*
	Briefly, the Physical Activity Guidelines for Older Adults was not appealing to the women in both focus groups because of the difficult language, technical terminology and information overload.	
	The “Be Active Your Way” was considered a mid-point in regarding preference between the three because it brings a compact message and it is more visual; despite some participants still recognize the terminology used as challenging.	**Be Active Your Way:** *“… It’s a little bit more condensed than the CDC paper and it doesn’t give you what the, all the wordings that the CDC paper does… it’s a little bit easier to read…” (66 years - Inactive)*
	At the other end the “Exercise and Physical Activity” brochure, language was only mentioned in a positive context.	*“… It’s more colorful, it has perked my interest. I’m a visual learner… I like just the bar on the bottom that says, “Be active, healthy, and happy”. I think those are active words… I want to be active, healthy, and happy… (66 years - active)*
		**Exercise and Physical Activity: Your Everyday Guide:**
		*“… I think that the instructions for the exercises are easy to read… they basically word it easy… and them of course having the illustration, kind of step by step, helps because you can actually see a picture and associated with the instructions you have… I like it because they have actually broken it down… they go in-depth, thinking about beyond a certain class of people… and make them want to read…” (65 years - Active)*
2. It does not reflect us	This theme represents the concern of the women from both focus groups regarding the examples of activities used in the materials. Women from both groups expressed their concern that examples of activities provided in the materials do not reflect the reality of the African American population.	*“…for black women… there were restrictions on the physical activity for black Americans… You couldn’t even go swimming in the pool. You were restricted. You certainly couldn’t hang out at the track in the high school and walk around the track at a high school, and be suspect. You couldn’t go to a public beach and swim…” (65 years - Active)*
		*“… it has a lot to do with economics… looking at these lists* [examples of activities provided in the materials for moderate and vigorous intensity] *to me I still keep going back to the black* versus *white, whereas these are not the things we have thought of all of our entire lives.” (66 years - Inactive)*

Materials included in the focus groups discussions, Exercise and Physical Activity: Your Everyday Guide; The Physical Activity Guidelines for Older Adults; and Be Active Your Way: A Guide for Adults

Although level of knowledge *per se* does not translate into sustained behavior [42], knowledge is considered an individual determinant that may have an influence in ones behaviors [9, 43]. An AARP study showed that although knowledge levels may be high among older adults, action upon knowledge is often very low. Moreover, the report identified that imagery and tone are very important in motivating people to be physically active. Accordingly, people over the age of 50 years are motivated by images they can relate to, not by elite senior athletes that make them feel discouraged and overwhelmed, the same way that a confrontational or critical “get off the couch” approach is often not effective. The report highlighted the fact that the majority of the seniors want more information about how to exercise safely, stay motivated, and set realistic goals [44]. The findings of the report corroborate the findings of the present study. Participants’ voices revealed that many of the examples used in the materials fail to reflect the reality of older African Americans (refer to Table 4 – theme 2). The legacy of segregation, racism, discrimination and factors such as economic concerns not only help explain some of the health inequities in terms of chronic diseases [45, 46] but may also be responsible for limiting African Americans from taking part in many of the examples described in the disseminated materials. As mentioned by a participant, historically these women have not been exposed to most of the examples provided in the brochures. This is consistent with the findings of a recent literature review [9] in which a lack of physically active role models among African Americans was found to be a barrier for PA in this population.

The findings from this pilot study suggest that materials used to educate and motivate the general public towards PA need to be modified to better speak to older AAW, especially those who are inactive and have difficulty in building PA into their daily lives. These adjustments not only apply to the level of language but also to the cultural relevance of the material. Identifying, recognizing and critically addressing the concerns raised in the current study is a first and a needed step towards developing comprehensive culturally sensitive materials to help improve PA level and consequently to enhancing health and quality of life in this population.

Our findings provide important information that can help to elaborate future, more culturally sensitive, messages aimed at promoting PA within this vulnerable population. Researchers have noted the importance of matching the cultural characteristics of minority populations with public health interventions in order to enhance receptivity, acceptance and salience of health information and programs. It is believed that belief systems, religious and cultural values, life experiences and group identity work as powerful filters through which information is received [23, 47]. Studies have shown that generating customized health messages/materials at the individual level is effective in health [28] and vital step towards promoting positive health behavior change practice [28, 48, 49]. A meta-analysis conducted by Noar and colleagues [28] provided evidence of the effectiveness of tailoring health behavior change messages and suggested different factors that appear to moderate the effects of tailoring. The authors concluded that interventions employing pamphlets, newsletters, or magazines including visual elements are among the strongest tailored health behavior change approaches. Such a conclusion is in line with the findings of the current study. Moreover, a literature review [48] found that in 10 out of 12 studies included in the review, evaluating message tailoring, tailored messages resulted in greater PA than more controlled and general messages.

Although tailoring messages is important, it is also important to create mechanisms for a message to reach the targeted population. The internet is often proposed as a channel by which to disseminate health information. A recent report showed that the use of internet is increasing among older adults of different race groups including African Americans [50]. However, there are still a large proportion of this population that accesses the internet infrequently or not at all [51] and a more comprehensive strategy is needed to reach this population. Future studies should explore possible ways to deliver healthy lifestyle messages in an effective way for older African American women and other vulnerable populations and to evaluate the impact of this process on PA and health outcomes. During the focus group discussions, none of the women reported having ever seen any of the materials. This suggests that there is a gap between developing and producing the materials and disseminating them in such a way as to reach the desired populations.

This study provides useful information for public health professionals on this under-researched issue and this vulnerable population. The inclusion of both active and inactive women in the focus group discussions may be considered strength of this study. Although important, our findings must be carefully interpreted due to some limitations. Because this was a pilot study, we employed a relatively small number of participants, who had fairly high income and education levels. Additionally, because we only collected information from 10 women, we did not know if saturation was reached. Together, the above points may reduce the transferability of the findings to the broader community of older African American women. In fact, the transferability of the results may be compromised even within the specific geographic location where the study was conducted. The perceptions of older AAW regarding the materials may have been different even within the same geographical community if a different sampling procedure had been used to select the participants. Future studies should try to duplicate these findings using different methods for sampling older AAW to increase our understanding on PA promotion in this population. Currently, a very low rate of African Americans, especially older AAW engage in sufficient PA to achieve health benefits. As noted by Williams and Jackson [46] health and health disparities are embedded in larger historical, geographic, sociocultural, economic, and political context. Therefore, changes in a broad range of public policies are needed to effectively address these systemic and ongoing disparities.

## Conclusions

The current study explored perceptions and reactions of older AAW to widely disseminated public information pertaining to PA promotion and aging. The analysis revealed some shortcomings in public health materials aimed to promote PA in the adult and older adult population not only in terms of language but also in terms of visual imagery. Together, these factors may be working as a barrier that detracts older AAW from the understanding and appreciation of the content provided in the materials. Since these informational materials are used to inform the general public about PA and exercise and to empower them through knowledge, the purpose may not be being achieved. It strongly suggest that materials used to educate and motivate the general public towards PA need to be modified to better speak to older AAW, especially those who are inactive and have difficulty in building PA into their daily lives.

## Abbreviations

AAW: African American woman
BMI: Body mass index
CDC: Centers for disease control and prevention
MVPA: Moderate to vigorous physical activity
PA: Physical activity
